# The relation between radiographic manifestation and clinical characteristics of congenital radioulnar synostosis in children: A retrospective study from multiple centers

**DOI:** 10.3389/fped.2023.1117060

**Published:** 2023-03-03

**Authors:** Pan Hong, Wei Tan, Wei-Zheng Zhou, Yu Zheng, Jin Li, PengFei Zheng, Xin Tang

**Affiliations:** ^1^ Department of Orthopaedic Surgery, Union Hospital, Tongji Medical College, Huazhong University of Science and Technology, Wuhan, China; ^2^ Department of Pediatric Orthopedic, Center for Orthopedic Surgery, The Third Affiliated Hospital of Southern Medical University, Guangzhou, China; ^3^ Department of Pediatric Orthopaedics, Shengjing Hospital of China Medical University, Shenyang City, China; ^4^ Basic Medical School, Tongji Medical College, Huazhong University of Science and Technology, Wuhan, China; ^5^ Department of Orthopaedic Surgery, Children's Hospital of Nanjing Medical University, Nanjing, China

**Keywords:** congenital radioulnar synostosis, pronation, ankylosis, classification, radiographic manifestation

## Abstract

**Objective:**

To review the radiographic manifestation and clinical appearance of children with congenital radioulnar synostosis (CRUS) retrospectively.

**Study design:**

Retrospective cohort study of children with CRUS from multiple medical centers.

**Results:**

A total of 329 patients (male 259, female 70) with an average age of 5.4 years (0.5–16 years old), were included in this study. In particular, 145 patients (145/329, 44.1%) demonstrated bilateral involvement, and 184 patients (left 123, right 61) demonstrated unilateral involvement. As for Clear and Omery (C&O) classification, most patients belonged to Type III, and then followed by Type IV. As for Chinese Multi-center Pediatric Orthopedic Study Group (CMPOS) classification, most patients belonged to Type III, and then followed by Type II and Type I. In C&O Type III, 92.03% patients demonstrated severe pronation. According to CMPOS classification, 92.98% Type I patients demonstrated neutral to mild pronation, 72.17% Type II patients demonstrated moderate pronation, and 92.03% Type III patients demonstrated severe pronation. The age distribution showed no significant difference between C&O Type II and IV (*P* = 0.96); the pronation ankylosis severity showed no significant difference between C&O Type II and IV (*P* = 0.387).

**Conclusion:**

Although CRUS is a rare forearm deformity, there are certain relation between radiographic manifestation and clinical forearm functional restriction. CRUS patients of C&O or CMPOS Type III classification might suffer severe pronation deformity and warrant early intervention.

## Introduction

Congenital radioulnar synostosis (CRUS) is an abnormal condition at the proximal radius and the ulna, with restricted forearm rotation as prominent features (1). The clinical characteristics of fixed forearm deformity varies across different patients, from neutral position to severe pronation (2). The functional disability depends on the fixed position of forearms, bilaterality, and social practice in different cultural backgrounds (3). Usually, x-ray of forearm should suffice for the diagnosis of this condition (1). Because of its rarity and the diversity of fixed position, there is no consensus on the treatment strategy (4, 5). So far, multiple surgical techniques and physiotherapy have been reported to be beneficial for this condition with improved life quality (6–9). However, the indication for surgery remains unclear and the postoperative outcomes remains mixed (1, 2).

Most investigations on CRUS were case reports or small cohorts. Sachar et al. reviewed the largest cohort study so far, with 350 cases in total (10). However, he merely reviewed these cases without in-depth analysis. Until present day, the literature has witnessed the publications of 616 cases in CRUS (1, 8). However, there existed no report on the correlation between radiographic manifestation and clinical function.

Nowadays, the widely adopted classification was proposed by Cleary and Omer in 1985 (11). Cleary & Omer classification (C&O classification) consists of 4 types based on the appearance of the synostosis and status of radial head on x-ray. However, because of the small cohort, the relation between C&O classification and clinical manifestation was not revealed clearly (11). In the past, supinator was thought to be absent in certain patients, which was repudiated by Li Jin et al. (12). Recently, a novel classification system based on x-ray and magnetic resonance imaging (MRI) was proposed by Chinese Multi-center Pediatric Orthopedic Study Group (CMPOS) (12). The supinator was taken into consideration in this novel anatomical classification and all patients demonstrated the presence of supinator in the MRI. However, there was still no elucidation on the relationship between radiographic classification and clinical manifestation. Therefore, a retrospective study from multiple medical centers with a large cohort of pediatric patients diagnosed as CRUS was performed.

## Methods

Patients diagnosed as CRUS were reviewed retrospectively from four high-volume geographically separated medical centers. Basic information were collected anonymously and centrally with written informed consent signed by legal guardians of the patients.

The inclusion criteria were: (1) younger than 18 years old at the time of diagnosis; (2) without previous surgery or instrumentation in the affected limb; (3) without concurrent syndromic malformation or deformity of the ipsilateral upper extremity. The exclusion criteria were: (1) concomitant neuromuscular disease; (2) incomplete medical records; (3) inconsistent diagnosis between two senior attending physicians; (4) patients with complicated syndromic manifestation. This study was approved by the ethnics committee of authors’ institutes.

All the radiographic data were extracted from institutes database and reviewed by two senior attending physicians. The types of x-ray manifestations were recorded according to the C&O and CMPOS classification if their results were consistent, otherwise a third observer would check and made the final decision. Clinical manifestations were extracted from institutes database and recorded by two physicians. The observers were not involved in the authorship of this research.

**Figure 1:**
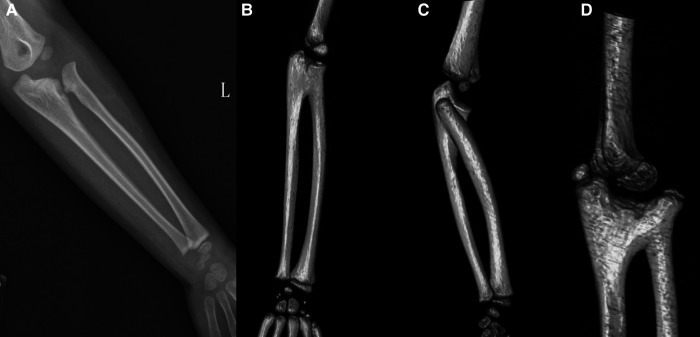
X-ray or CT reconstruction of forearm deformities. (**A**) C & O Type I; CMPOS Type I. (**B**) C & O Type II; CMPOS Type II. (**C**) C & O Type III; CMPOS Type III. (**D**) C & O Type IV; CMPOS Type II.

C&O classification consists of four types: (I) fibrous ankylosis with normal radial head; (II) osseous synostosis with normal radial head; (III) osseous synostosis with posteriorly dislocated and hypoplastic radial head; (IV) pseudo-synostosis and anteriorly dislocated, mushroom-shaped radial head (11). CMPOS classification consists of three type: (I) fibrous pseudo-synostosis with any shape of the radial head; (II) osseous synostosis with or without dislocated radial head; (III) radiographic radial head unobservable and osseous synostosis with the ulna (12) (see Figure 1).

One-way ANOVA test, Student's *t* test and SNK-q test were conducted. Statistical analysis was performed using the IBM SPSS statistics version 20 software (SPSS Inc., Chicago, Illinois).

## Results

A total of 329 patients (male 259, female 70) with an average age of 5.4 years (0.5–16 years old), were included in this study. The age was the patient firstly diagnosed as CRUS in the clinic. In particular, 145 patients (145/329, 44.1%) demonstrated bilateral involvement, and 184 patients (left 123, right 61) demonstrated unilateral involvement. Only three patients with fixed supination deformity were included, and the rest demonstrated pronation deformity. As for C&O classification, most patients belonged to Type III, and then followed by Type IV. As for CMPOS classification, most patients belonged to Type III, and then followed by Type II and Type I (see Table 1).

**Table 1:** Demographics information of patients distribution in different classification.

Classification	Side	Gender	Age of diagnosis (mean ± S)	Unilateral or bilateral	Total
	L/R	M/F	L/R	U/B	
**C&O**					
I	14/11	20/5	5.28 ± 3.53	23/2	25
II	18/26	33/11	6.35 ± 3.65	42/2	44
III	171/130	230/71	4.66 ± 2.64	226/75	301
IV	65/39	95/9	6.62 ± 3.61	91/13	104
**CMPOS**					
I	37/20	50/7	5.26 ± 3.15	51/6	57
II	59/56	97/18	6.82 ± 3.65	104/11	115
III	171/131	231/71	4.70 ± 2.70	226/76	302

As shown in Table 2, according to C&O classification, 88.5% Type I and 45.71% Type IV patients demonstrated neutral to mild pronation. However, 68.17% Type II and 55.33% Type IV patients demonstrated moderate pronation. In C&O Type III, 92.03% patients demonstrated severe pronation. According to CMPOS classification, 92.98% Type I patients demonstrated neutral to mild pronation, 72.17% Type II patients demonstrated moderate pronation, and 92.03% Type III patients demonstrated severe pronation.

**Table 2:** Type of patients distribution in different pronation degree.

Classification/(Percentage)	Supination	Neutral to mild pronation	Moderate pronation	Severe pronation	Average degree (mean ± S)
	<0°	0°–20°	21°–59°	≥60°	
**C&O**					
I	1 (3.85%)	23 (88.5%)	1 (3.85%)	1 (3.85%)	9.29 ± 13.85
II	2 (4.55%)	10 (22.73%)	30 (68.17%)	2 (4.55%)	25.68 ± 17.51
III	0 (0.00%)	1 (0.33%)	23 (7.64%)	277 (92.03%)	62.81 ± 7.79
IV	1 (0.95%)	48 (45.71%)	56 (55.33%)	0 (0.00%)	22.79 ± 13.01
**CMPOS**					
I	1 (1.75%)	53 (92.98%)	2 (3.51%)	1 (1.75%)	9.65 ± 13.20
II	3 (2.61%)	27 (23.49%)	83 (72.17%)	2 (1.74%)	27.28 ± 13.09
III	0 (0.00%)	0 (0.00%)	24 (7.97%)	278 (92.03%)	62.82 ± 7.69

The age distribution showed no significant difference between C&O Type II and IV (*P* = 0.96); The pronation ankylosis severity showed no significant difference between C&O Type II and IV (*P* = 0.387, see Table 3).

**Table 3:** T-test on the distribution of II and IV patients in C&O classification.

Classification	Supination	Neutral to mild pronation	Moderate pronation	Severe pronation	*P* value
	<0°	0°–20°	21°–59°	≥60°	
II	2	10	30	2	0.387019678
IV	1	48	56	0	

As shown in Table 4, in the 145 patients with bilateral involvement 38.62% patients demonstrated identical pronation degrees, while 31.03% patients demonstrated more than 30 degrees difference between two arms. As for C&O classification, 92 patients (63.4%) demonstrated the same category; as for CMPOS classification, 93 (64.1%) showed the same category.

**Table 4:** Differences in the right and left hands pronation degree in patients with bilateral involvement.

	No difference	Similar severity I	Similar severity II	Similar severity III	Obvious differences
	Degrees difference = 0°	0° < Degrees difference < 10°	10° ≤ Degrees difference < 20°	20° ≤ Degrees difference < 30°	Degrees difference ≥ 30°
No./(Percentage)	56 (38.62%)	20 (13.79%)	16 (11.03%)	8 (5.52%)	45 (31.03%)

## Discussion

To the best of our knowledge, this study was the largest cohort on CRUS highlighting the association between radiographic manifestation and clinical forearm functional restriction. In this study, correlation between the pronation ankylosis and radiographic manifestation was shown in our results. Most patients in C&O and CMPOS Type III demonstrated severe pronation deformity (>60 degrees), while patients in other types demonstrated sparse distribution without normality.

Several previous studies focusing on the surgical intervention for CRUS highlighted the fixated pronation deformity as one important criterion for surgery (13–16), but the latest meta-analysis in 2022 still reached no consensus on the severity of pronation and the necessity of surgery (13). Because of its rarity, most case series did not stress on the correlation of x-ray manifestation and forearm pronation deformity.

Many surgical techniques have been reported, including derotation and mobilization surgeries (14–16). However, the optimal surgical technique remains controversial, and the reported clinical outcomes after surgery were inconsistent. Until now, there was no clinical classification guiding treatment algorithm. Besides, there was no classification reflecting clinical function of forearms. For patients with neutral or mild supination deformity, surgical intervention is not necessary. Physiotherapy seemed to be beneficial for patients with moderate rotation restriction (9). However, the criteria for surgery remains unclear. Ankylosis pronation over 60 degrees has been proposed as a criterion for operation (3–5). Patients with bilateral involvement should be carefully evaluated for daily activities restriction and necessity for surgery (1–5). Certain authors recommended surgeries for patients with bilateral pronation over 45 degrees (4, 5). Pronation severity as an important parameter has been mentioned in all publications on operative choices. However, there was no clear correlation between the pronation ankylosis and the necessity of surgery (1–7), even 20 degrees pronation was reported for surgery (14). The optimal age of surgery remains to be investigated, 1.5–9 years old have been reported in the literature (1, 2, 14).

Currently, there is no actionable classification for treatment algorithm. C&O classification is widely adopted in academic publications, but it did not reflect the severity of deformity regarding the forearm functional restriction (11). Li Jin et al. confirmed the presence of supinator in all patients with CRUS in their study (12). However, the novel CMPOS classification is still an anatomical and radiographic classification without direct reflection of the forearm functional restriction (12). Patients in type II and IV demonstrated similar pronation ankylosis degrees without chronological relevance. In this study, the manifestation of C&O II and IV was similar in age and ankylosis pronation severity, which corroborated the fusion of C&O classification II and IV as CMPOS type II (12).

The results of this study showed that most of the patients belonged to type III with prominent pronation deformity, partly because of its severity of fixed pronation leading to earlier detection. Type III are similar in C&O and CMPOS classification, as it demonstrates osseous synostosis with a hypoplastic and posteriorly dislocated radial head (11). Therefore, patients with type III deformity with severe pronation might be beneficial from early surgery. Moreover, patients with bilateral involvement did not demonstrate the same severity in both forearms, consistent with earlier publications (1, 2).

There are certain limitations in our study. Firstly, it is a retrospective study without long term follow-up. Secondly, our data did not demonstrate normal distribution because of relatively small sample size. Further investigation with longer follow-up and advanced radiological examinations including CT scan or MRI might be warranted to explore more meaningful facts about this disease.

## Conclusion

Although CRUS is a rare forearm deformity, there are certain relations between radiographic manifestations and clinical forearm functional restriction. CRUS patients of C&O or CMPOS Type III classification might suffer severe pronation deformity and warrant early intervention.

## Data Availability

The raw data supporting the conclusions of this article will be made available by the authors, without undue reservation.
